# The Daily Mile: What factors are associated with its implementation success?

**DOI:** 10.1371/journal.pone.0204988

**Published:** 2018-10-04

**Authors:** Gemma C. Ryde, Josephine N. Booth, Naomi E. Brooks, Ross A. Chesham, Colin N. Moran, Trish Gorely

**Affiliations:** 1 Faculty of Health Sciences and Sport, University of Stirling, Stirling, Scotland, United Kingdom; 2 Institute of Education, Community and Society, University of Edinburgh, Edinburgh, Scotland, United Kingdom; 3 Department of Nursing, University of the Highlands and Islands, Inverness, Scotland, United Kingdom; University of New Mexico, UNITED STATES

## Abstract

**Background:**

Despite the known benefits of a physically active lifestyle, there are few examples of interventions that have been successfully implemented at a population level over a long period of time. One such example is The Daily Mile, a school based physical activity initiative, where a teacher takes their class out daily during class time for a short bout of ambulatory activity. At one school, this activity appears has been sustained over a long period (6 years), has the whole school participating and is now incorporated into its daily routine. The aim of this paper was to understand how The Daily Mile was implemented in primary schools and to assess factors associated with its successful implementation.

**Methods:**

Semi-structured interviews with school staff who had a significant role in implementing The Daily Mile were conducted at four primary schools in central Scotland. Interviews were digitally recorded and transcribed verbatim. Data were analysed using thematic analysis and descriptive analysis and interpretation of data undertaken. Details regarding the school grounds and facilities were also noted during the interviews.

**Results:**

Having simple core intervention components, flexible delivery that supports teacher autonomy and being adaptable to suit the specific primary school context appear to be key aspects of The Daily Mile that are related to its implementation success. Other factors relating to how The Daily Mile was developed, trialled and rolled out might also have contributed towards its successful implementation.

**Conclusion:**

The Daily Mile appears to have several factors which may relate to its implementation success. These are important considerations for others looking to implement The Daily Mile effectively in their primary school or in other contexts.

## Introduction

Physical inactivity is a global issue with both adults and children in many countries not active enough to benefit their health. [1] Physical inactivity is related to negative health outcomes including cardiovascular disease, diabetes, obesity, and depression, with regular physical activity beneficial in both the prevention and treatment of many conditions. [2] However, despite the known benefits of a physically active lifestyle how to increase activity at a population level remains a key question. Many interventions are developed in a research context, appear to work in controlled conditions but do not translate well into less controlled, real life situations. [3, 4] Implementation evaluations are rarely conducted alongside these interventions and therefore a challenge remains in understanding how to translate and scale up seemingly effective interventions. [4]

An alternative approach to developing an intervention in a research context is instead to evaluate existing interventions that are developed by those already working within complex, real life contexts. One such example of an intervention developed in a real life context is The Daily Mile. Originally conceived by a primary school in central Scotland in 2012, The Daily Mile was developed to address a perceived lack of fitness in primary school children (children aged five to twelve). The Daily Mile was seen as a pragmatic solution to this issue and was developed without specific reference to psychological theories of motivation or behaviour change or implementation frameworks or concepts. The intervention consists of teachers leading their class in an outdoor walk, jog or run for approximately 15 minutes every day during class time. As the approximate distance covered within this time was around a mile, the endeavour was named ‘The Daily Mile’. The Daily Mile appears to be addressing its objectives with preliminary pilot data suggesting that it may improve fitness, physical activity, body composition and have positive changes on classroom behaviour. [5]

At this first school, The Daily Mile has been sustained over a long period (6 years), has the whole school participating and is now incorporated into its daily routine. The Daily Mile has therefore gained much attention in the media and from other schools wishing to adopt this approach. In Scotland, the Government have issued a statement saying that they “are encouraging all schools to consider implementing the scheme (The Daily Mile) or develop their own physical activity initiatives” and supported the idea of a Daily Mile Nation in their 2016 manifesto. [6] Subsequently, this intervention has been scaled-up with over 770 primary schools in Scotland and 3600 worldwide registered as implementing The Daily Mile. [7] Based on this widespread roll-out and adoption it could be suggested that The Daily Mile is an implementation success.

To date, there is no evidence to support why The Daily Mile might be an implementation success and what others wishing to adopt the initiative should consider. Whilst numerous systematic reviews report on the outcomes of interventions to promote physical activity in school settings, few have looked at implementation considerations.[4, 8–12] Naylor et al. (2015) conducted a systematic review to assess factors that may influence the implementation of school based physical activity interventions in six to 18 year olds.[8] They reported time constraints as the most commonly noted factor linked to implementation, with interventions that competed for too much time in the curriculum or that were very time intensive to deliver being less successfully implemented. Other factors noted in this review as being associated with implementation success included a supportive school environment, perceived appropriateness of the intervention and teachers self-efficacy to deliver the intervention. However, the extent to which these or other factors are evidenced within the implementation of The Daily Mile have not yet been explored.

The aim of this paper was therefore to understand how The Daily Mile was implemented in primary schools and to assess factors associated with its successful implementation.

## Methods

### Recruitment

The current study was part of a broader research project investigating the benefits of taking part in The Daily Mile. [5] Four state primary schools in central Scotland were identified through this existing research project and with key contacts at these school informed about the study. Two of the schools were thought to be implementing The Daily Mile successfully with the other two thought to have found implementation more challenging. One of the schools was the primary school were The Daily Mile was first developed. The key contacts then suggested a representative who played a significant role in The Daily Mile implementation who were then invited to participate in the study. These individuals often gained the perspective of their colleagues prior to interview and provided an overview of The Daily Mile at their school. In addition, the head teacher who conceived The Daily Mile at the original primary school (now retired) was contacted through the broader research project and invited to take part in the study. Interviews took place from December 2015 till March 2016. All interviewees provided informed consent and ethical approval was obtained from the University of Stirling, School of Sport Research Ethics Committee (reference number 760). Approval was also obtained from the Director of Children, Young People and Education at Stirling Council, Scotland.

### Interviews

Qualitative interviews were selected as the most appropriate method to address the research questions and initial theories were developed around which elements of The Daily Mile were related to successful implementation.[13] Three semi-structured interview schedules were then developed. One for the retired head teacher who initiated The Daily Mile, one for the schools who reported currently having The Daily Mile and one for schools for whom implementation had proved more challenging. The schedules were developed to address the key research questions but contained some variation depending on whether the implementation of The Daily Mile had been considered successful or not. Questions included; what is a typical day at their school; why they started The Daily Mile; how it was implemented at the school; what barriers they faced; and its perceived benefits. Initial interview schedules were developed by GR and reviewed and edited by TG. GR conducted all interviews and kept detailed field notes pertaining to the schools context (e.g., details about the school grounds, number of children, number of classes, school postcode). Initial analytical thoughts were written up after each interview as is standard practice in qualitative interviews.[13, 14]

### Descriptive data

Postcodes of the schools were used to assess deprivation and rurality. Deprivation was assessed using the Scottish Index of Multiple Deprivation (SIMD). [15] The SIMD assesses levels of income, employment, health, education, geographic access, housing and crime in postcode areas, which are then categorised into quintiles from the highest areas of deprivation (SIMD 1) to the lowest (SIMD 5). Rurality was assessed with The Scottish Government Six Fold Urban Rural Classification. [16] The six classifications are: large urban areas (settlements of 125,000 or more people), other urban areas (settlements of 10,0000 to 124,999 people), accessible small town (settlements of 3,000 to 9,999 people within 30 minutes’ drive of a settlement of 10,000 or more), remote small town (settlements of 3,000 to 9,999 people with a drive time of over 30 minutes’ to a settlement of 10,000 or more), accessible rural town (areas with a population of less than 3,000 people, and within a 30 minute drive time of a settlement of 10,000 or more) and remote rural (areas with a population of less than 3,000 people, and with a drive time of over 30 minutes’ to a settlement of 10,000 or more).

### Data analysis

Interview discussions were digitally recorded and transcribed verbatim and field notes written up after each interview. Throughout the data collection process, data were analysed using the constant-comparative technique.[17] GR reviewed and compared interview transcripts and field notes after each interview which enabled the identification of emergent themes for exploration in following interviews. Subsequent data management and analysis was approached using thematic analysis.[18] Familiarisation with data enabled construction of first level coding informed by 1) the research questions underpinning the study, 2) topics and issues introduced by researchers during the interviews and 3) recurring themes emerging from interviews discussions.[19] In order to ensure consistency and rigor, double coding was carried by TG.[19] In addition, all codes were reviewed and verified by an independent researcher who was not part of the research team. Descriptive analysis and interpretation of qualitative data were then undertaken. Due to the number of schools involved in the study, quantitative data (number of children and classes at each school) are presented as raw data in text.

## Results

A brief description of the participants and schools involved in the study is provided at the outset of the results. The subsequent section highlights the overarching themes from the qualitative data.

### Participants and schools

Six participants from four primary schools took part in the study. Participant’s included three current head teachers, one teacher, one classroom assistant and the retired head teacher who conceived the original Daily Mile. All participants were female and confirmed they played a significant role in either the initial implementation or the current delivery of The Daily Mile. School size varied with schools having 170, 334, 360 and 540 children. The number of classes at each school also varied with schools having 7, 12, 13 or 16 classes. Three schools were classified as being within “other urban areas” and one as within an “accessible small town”. Two schools were in areas of high deprivation (SIMD 1) and two were in areas of low deprivation (SIMD 5). During the interview process it transpired that one school that was thought to have difficulties implementing The Daily Mile was actually implementing it as expected, therefore, three of the four schools were reported to be currently delivering The Daily Mile and had done so for between three months to six years.

### Themes

#### Intervention development, trialling and roll out

The Daily Mile began in one class in a large operational primary school in Central Scotland. It was described as having started as a result of a conversation between a teacher and an external activity facilitator about the children’s lack of fitness. This perceived lack of fitness was confirmed with physical education staff, and working with their teacher, one class tested their fitness by seeing how far they could run without needing to stop. The teacher described how she then spoke with the children who acknowledged that there was a problem with their fitness and together they came up with a four week program to get outside and walk, jog or run for 15 minutes every day. After the four weeks the children and teacher noticed a change in the distance they could run.

*“…he said*, *the children are not fit*, *and I*, *you know*, *I knew they weren’t fit*, *so I spoke to the*, *the gym teacher and said “am I*, *are we right*, *are the children not fit*?,*” and she said “the children are exhausted by the warm-up in PE*, *and they have to recover from the little warm-up that we do*, *and they can't access PE properly*, *yes they're unfit*.*”* [P6]

The participant noted that this story and perceived change was communicated with other teachers who were encouraged and supported by the school to do something similar with their class if they chose to. She describes how concerns teachers might have about how The Daily Mile would work were discussed collectively and problem solved. The Daily Mile slowly began to spread throughout the school and by the summer that same year, all classes were involved.

The participant described how the idea of The Daily Mile and the anecdotal results were then communicated with other schools in the area, largely through herself who became an advocate for the initiative. She noted that the media also reported the story and it received a great level of publicity nationally. Other schools then began to implement their own Daily Mile. In the schools interviewed for this study the head teachers and whole school decided to support the initiative and typically every class took part. As with the original Daily Mile, after the decision was taken by these schools to implement The Daily Mile, participants reported consultations between teaching colleagues as to how The Daily Mile could work in their context.

*“… so I think the drivers are the team–the leadership team–the children … and the teachers*, *you know it's*, *it's working altogether …”* [P3]

#### Simple core intervention components

Three core intervention components were identified relating to the type, duration and frequency of the activity with the simplicity of these and suitability to the primary school context likely to be related to its implementation success.

According to participants, primary schools predominantly chose to implement walking, jogging or running as the type of activity, as per the original Daily Mile concept.

*“…it catered for all abilities … the ones that maybe are a bit slower*, *'cause you'll*, *you'll get out there and some o' the kids will just run*, *run round and they’ll*, *they want to know how many laps they can do*, *and whereas you've got other ones that are happy just to walk …”* [P4].

At one school the participant reported there were concerns about a small and difficult shaped playground and it was decided to change The Daily Mile into the Daily Skip, with the view that “*the children were having that physical activity*, *getting similar kinds of benefits but it was within a confined space”* [P2]. Skipping was chosen over walking as it could be completed in the small playground at the school and would keep the children visible and on site unlike The Daily Mile which would have to be completed offsite. The participant reported that The Daily Skip proved very challenging for a number of reasons including the choice and cost of equipment (different lengths needed, children preferring different types of ropes), the logistics of managing the skipping ropes (distribution, drying ropes when wet), and the skill required to skip. They highlighted how these challenges which move away from the original simple design, plus other demands from within the curriculum meant that The Daily Skip was only happening sporadically, if at all.

*“So at first it*, *obviously it's [skipping] quite an expensive thing as well*, *because again you need skipping ropes*, *and because every class has kind of been encouraged to do it when it suits them*, *you know you needed multiple sets of them …and when you're out skipping in the rain with skipping ropes they get wet*, *they get sodden*, *they get dirty*, *they get harder to manipulate and to move*, *particularly for the younger children*, *because my class*, *some of them are*, *in fact the biggest majority can't actually skip*, *they can't rotate the rope round and skip continuously the way that other classes can*, *so we're kinda skipping over a rope and it's*, *it's just difficult 'cause you're skipping into puddles*, *you're skipping in wetness… so there's been lots of things that have proved very difficult …”* [P2]

The duration of the activity at all schools was 15 minutes. One participant noted that although it is called the Daily *Mile* the intervention is actually time (15 minutes door to door) not distance based. It was said that if the children were running then they achieved approximately one mile during this time. This amount of time was also perceived as being short enough not to interfere too much with the school day. During the interview process, one of the schools who was thought not to be completing The Daily Mile, described their “unsuccessful” attempts at implementing it within their context. Through this description it emerged that they were in fact delivering The Daily Mile but were not aware they were. Specifically, the participant discussed how the school initially had the children completing an actual mile. However, they then noted that for reasons of time (it took too long for some children to complete an actual mile) and motivation (a mile was a long way for some children), it was felt that the activity was not suitable for long term engagement and was not incorporated into the school’s routine. Subsequent to this, the school adapted their approach to 15 minutes of activity (which is what The Daily Mile was intended to be) and this was reported to be more feasible. The Daily Mile continues to be completed at this school in this way highlighting the importance of the short duration of The Daily Mile.

*“…it shouldn’t take any longer than about 15 minutes in total*, *‘cause otherwise we’re looking at quite a chunk of time in their learning…it isn’t a mile that’s measured out*, *it’s a time period*, *it’s how far you can go within that period of time*.*”* [P1]

Other factors that increased the time taken to complete The Daily Mile were also reported to be considerations for implementation. Whilst The Daily Mile was described as not requiring any specific equipment or kit, one participant reported that some children liked to change their shoes before completing The Daily Mile and that sometimes jackets were necessary if it was cold or wet. At this school the jackets were kept in the classrooms and therefore the participant suggested that changing was not seen as an issue as these could be accessed quickly.

*“Some of the children like to change into trainers*, *and you know we give them that couple of minutes just*, *they know quickly that it’s part of the routine*, *they don’t need to come and ask*, *they just go and do it*.*”* [P3].

However, in another school the participant described how the cloakrooms were away from the classroom and if jackets and shoe changes were needed then this added to the time taken for The Daily Mile. Whilst this participant did not note that this had an immediate effect on implementation and it didn’t affect all classes, it was an issue raised by teachers.

The frequency of The Daily Mile was typically less than five times per week and rarely occurred on a daily bases. While participants stated that the school and their colleagues had aspirations for The Daily Mile to occur every day, it typically took place on days with no scheduled physical education. The Daily Mile was therefore reported by participants to be occurring at least three times a week with days when there was already a scheduled activity such as physical education typically when it did not occur.

…*”so it's to make sure that they're getting some physical activity built in tae their day on the days that there's no physical education …so that would be three times’ a week”*. [P4]

#### Flexible delivery that supports teacher autonomy

All participants reported that The Daily Mile occurred during school time. However, they noted that the individual classroom teachers decided when in the day to take the children out allowing for teacher autonomy. Participants suggested that The Daily Mile was either timetabled in by the teacher to suit their plan for the day or taken at a point in the day when the children were flagging. One participant noted that availability of staff was an additional consideration as to when The Daily Mile occurred during the day. They commented that classes with children who had additional support needs often waited for a classroom assistant to be present in order to undertake The Daily Mile. It was noted that there were some teachers who choose to tag The Daily Mile onto an existing break because the children were already outside and in their outdoor attire resulting in less disruption over the day.

*“The Daily Mile sometimes will be timetabled there*, *sometimes it will not and the teachers will just go out when they feel …so it will vary day by day and even if it is up there for it to happen like halfway between break time and lunchtime*, *there's obviously that flexibility if something else is going on in the class*, *they might continue with that or if the teacher feels ‘och*, *we need a bit of a break now*, *let's go out*.*”* [P3]

*“…if they're out for their playtime it's easier for teachers to say you've got your outdoor stuff on*, *we’ll just go and do our Daily Mile*, *but if they've already had fifteen minutes of running around on a lunchbreak I really want them to come in*, *settle*, *do a bit of learning*, *go out and come back to their learning to see if it really has the impact*, *'cause for me it's about impact on learning …”* [P6]

#### Adaptable to suit the specific primary school context

Whilst participants described situations where changing core intervention components might have a negative effect on implementation, many aspects of The Daily Mile were reported to be adapted to make it work in their context. These were said to be largely related to the route and to suit the individual schools physical environment. However, even within this adaptability, participants would often describe a balance between adapting the Daily Mile to suit their context and making significant changes that might again negatively influence implementation.

Managing the usage of the route of an aspect of implementation that was adapted between schools. Two participants discussed that, due to space in the playground, there was need to schedule when specific year groups were able to go out (i.e. years 1–3 in the afternoon and the other classes in the morning). One participant noted that colleagues whose classrooms overlook the playground would look out the window and see whether the playground was clear before choosing to take their class out. Another said that there was a set direction to follow and when classes went out, they just joined into the flow. However, the exact time the classes go out within these periods was still at the discretion of the teachers retaining their autonomy.

*“…if they're out for their playtime it's easier for teachers to say you've got your outdoor stuff on*, *we’ll just go and do our Daily Mile*, *but if they've already had fifteen minutes of running around on a lunchbreak I really want them to come in*, *settle*, *do a bit of learning*, *go out and come back to their learning to see if it really has the impact*, *'cause for me it's about impact on learning …”* [P6]

The surface for the route was reported to be different between schools and even changed within schools due to the weather or for construction but participants suggested this did not affect implementation. For example, one participant reported that their school had an all-weather track as the route for The Daily Mile and due to construction work, the route temporarily changed to tarmac for a short period. During this time The Daily Mile still continued. Another participant noted that teachers would sometimes switch to using a grass area when it was dry. However, most described grass as being too wet, slippery or muddy to use for The Daily Mile.

*“… the grass is probably the least favourable option to run on [slight laugh] …em*, *you know*, *at the moment we're running on the tarmacked area and that's absolutely fine*, *it works really well*, *the children move from one running surface to another and they’ve never bothered at all*.*”* [*P*3]

Participants reported that the number of laps was adapted by schools to suit their playgrounds. Using laps was said to be linked to many important aspects of The Daily Mile including having no winners or losers, children counting laps to see their progression and being able to gather all children in quickly at the end. At one school with a small playground, there was a split level with stairs, narrow alleyways and ramps connecting the different areas. The participant from this school described the large number of laps that would have to be completed as “*almost like a prison sentence*” with children having to run around in circles in a confined area. In general, fewer laps were reported as more favourable.

*“We wouldn’t have the breadth and space to actually make that [laps] workable; it would be OK at the back playground but it would be very boring I think for the children*, *'cause they'd be walking in lines*: *back*, *forward*, *back*, *forward*, *and they would have to do that*, *what*, *I mean I don't know the maths*, *but I don't know how*, *they would have to do that*, *you know probably a*, *a good hundred times [slight laugh] or something* …” [P2]

The idea of balancing adaption whilst ensuring The Daily Mile integrity was also highlighted when participants discussed teachers taking part and the visibility of the children. Teachers taking part in The Daily Mile with the children was seen as favourable by most participants and occurred at the original school. They reported that teachers often used it as a time to connect with the children in a non-formal, classroom setting. However, visibility of the children whilst completing The Daily Mile was reported as more of a priority, with having the children out of sight a barrier to implementing The Daily Mile for the participant from the school which had a fragmented playground. In such circumstances when visibility was comprised participants’ suggested that teacher’s participation was sacrificed with teachers often choosing to remain in a fixed position to be able to keep sight of the children.

*“They [the teachers] can join in*, *some do*, *but if they’re needing to see the children at all points then they can’t…it depends on individual classes and you know the teachers*, *but the teachers would generally stand where they can see the whole class*.*”* [P3]

Participants described a similar notion in relation to keeping The Daily Mile onsite. At three schools, including the original school, participants reported that their routes were onsite. One participant discussed that their school had considered using an offsite location for The Daily Mile, however, they explained that this was not considered workable as it would add a level of logistics that was not feasible, such as needing more adults (teachers, classroom assistants or parents) to marshal the route. They also suggested a risk assessment would need to be carried out daily to ensure that nothing had altered on the route.

*“…we thought well can we take it outside of the school*?, *obviously we've got a local walk …and we could work out a mile route round about that*, *but to police that*, *it's all different gradients*, *it's all*, *obviously different areas*, *and for us to do that every day we’d have to have people*, *we’d have to have parents and staff out monitoring each different part because it's out in the community*, *and also you would have to be doing Risk Assessments every day*, *thinking about needles*, *dogs’ dirt*, *whatever it might be*, *but you know obviously there would be a lot of different factors to take into consideration*, *and we didn’t feel that that was going to be a viable option because the whole point of The Daily Mile as far as we were concerned was that it was done at a time that best suited a class teacher…”* [P2]

In addition to adaptations related to the physical environment, adaptations to suit children’s clothing were also noted by participants. A participant from a more affluent school noted that girls especially wore shoes which might not be suitable for running (ballet style pumps or shoes without shoelaces). In this situation they reported that the activity intensity was adapted and the children were encouraged to walk and not run.

*“…if children are wearing*, *you know*, *shoes like this then we would encourage them to*, *to walk fast*, *if they've got nothing else to change into*, *so it's that fast kind of you know*, *walking rather than running*, *where something might flop off their feet*, *you know…”* [P3]

#### Continuing the daily mile beyond implementation

Participants noted other factors during discussions which may not have been directly related to its initial implementation but could influence the longer term continuation of The Daily Mile within primary schools.

All participants mentioned The Daily Mile in reference to a busy curriculum. Participants noted that activities such as physical education, school plays and concerts were potential competition to The Daily Mile within the schools timetable. Some participants therefore reported that The Daily Mile was, on occasion, linked to the curriculum (e.g. maths) or to external events (e.g. Olympics, Commonwealth Games) as a way to not only enhance learning but to keep The Daily Mile current and relevant. It was reported that the original intention The Daily Mile was not that it be used to rehearse times tables for example as it should allow kids to turn off and enjoy being outside and that this concept should be maintained.

*“…they (the children) get excited about doing it if the teachers have come up with some ideas with the children about using the running*, *The Daily Mile to actually be part of their maths*, *or to be part of their topic work*, *so there's all that*, *you know*, *giving it a focus for a wee while*, *and that's not every single day of the year*, *that will maybe be for a part of the year and that's just to kind of give it a little boost*, *add some focus to us*, *and they enjoy that*, *yeah they enjoy it if they think they're running ‘The Daily Nile’ or doing different things like that*, *sort of tying it in with their*, *their class work”* [P3]

Participants also discussed monitoring classes’ participation in The Daily Mile to ensure it was actually occurring. The original Daily Mile was not monitored formally and participants suggested that over monitoring may hamper its success. This thought was reiterated by others who suggested that any monitoring should be light touch. One participant noted how their school informally monitored The Daily Mile through feedback from children and parents and general awareness of the head teacher of what was occurring at their school. Another reported that pupil councils or class representatives often provided feedback to the head teacher of when and how often The Daily Mile was occurring. However, this participant also suggested that too much deliberate enquiry about The Daily Mile via the children could damage teacher-pupil relationships and the need to ensure monitoring its usage didn’t have other unanticipated negative effects.

*“…in a school this size it would be very difficult to monitor*, *you know*, *and make sure everybody’s doing it … kinda thing … you know*, *how do you monitor when somebody’s got the freedom tae do it at any point in their day and on the days that they're not having physical education you know*, *what do you do*, *do you then turn the children in tae tell-tales and then what does that do tae the relationship between the children and the teacher*? *As far as I'm concerned you know*, *healthy*, *happy schools are about good relationships and you don’t want to be doing that*, *do you*?*”* [P4]

Although not directly referred to as inequalities by participants, some points raised related to disparities between pupils and how this could affect The Daily Mile. For example, the issue of appropriate footwear and clothing was raised by one participant whose school was in an area of high deprivation. They reported that the children would quite often come to school in clothing that was not appropriate for taking part in basic school activities making participation in The Daily Mile difficult. They said that some had shoes with holes in them or didn’t bring a jacket into school. They also suggest that poor footwear and lack of wet-weather clothing played into the schools decision as to whether to go out for The Daily Mile in wet conditions.

A key consideration noted by all those with The Daily Mile was that of the benefit teachers perceived from their class taking part which could contribute towards continuation of The Daily Mile. A sense that the children were re-energised and rejuvenated was noted by all as a perceived benefit of The Daily Mile. This emerged at all schools regardless of when in the day The Daily Mile was undertaken. At one school the participant mentioned that some children, typically those who had existing behavioural concerns, could become over stimulated as a result of taking part in The Daily Mile and took time to calm down once back in the classroom. However, on the whole, positive effects were most commonly reported even among children who were already perceived as being physically active. For example, in one school the participant suggested that many of the children were taking part in sporting activities and clubs outside of school hours. For these children, The Daily Mile was still reported to provide an instant boost in energy and improve concentration specifically during school hours. The desirability of these immediate benefits may reinforce teacher’s motivation to continue making time for The Daily Mile.

*“…it [The Daily Mile] can be a good energiser*, *so some of them [teachers] have said that the opportunity to get out and get moving and what have you*, *can be really good for energising and then re-focusing children …”*[P4]

## Discussion

The Daily Mile was developed at an operational primary school in central Scotland as a pragmatic solution to deal with the perceived lack of fitness of children. The aim of this paper was to understand how The Daily Mile was being implemented in primary schools and to assess potential factors associated with its successful implementation. From a sample of four primary schools, there are several potential factors relating to how The Daily Mile was develop, trialled and rolled out, its core intervention components and other factors relating to its delivery that could have positively influenced its implementation.

Whilst The Daily Mile was developed without reference to specific psychological theories or implementation frameworks and concepts, many of the intervention components and how it was delivered have come from a deep understanding of the primary school system and what would work in this context. The key factors related to its successful implementation as identified in this study are therefore largely reflected in implementation literature and include the simplicity of the core intervention components and adaptability of other intervention components to suit the specific primary school context [20]. Its core intervention components are intuitively simple with regards to duration, type of activity, and frequency and these are likely to be key to its success. Where The Daily Mile was interpreted as a literal mile and took more than 15 minutes, or it was decided to implement a more complex activity such as skipping, there were difficulties in implementation and the initiative was not sustained. Other intervention components such as the specifics of the route including its surface and how the route was managed were, with reason, adapted to suit each school context. The ability to adapt aspects of The Daily Mile are likely to have contributed positively towards its transferability to other primary schools, although as with the core components it appears to be important to retain the integrity of The Daily Mile where possible. This flexibility to adapt certain aspects of the intervention to suit the different primary schools context has also reported as an important component of other school based physical activity interventions. The Kids Marathon was a primary school-based participation challenge that gives children the opportunity to complete a marathon over a whole school year by running laps of a course 1–2 times per week in their lunchtimes. In their evaluation, Chalkley et al. suggest that contextual factors influenced how the Kids Marathon was implemented in different schools [21]. Schools may wish to consider what impact that changing the original, simple format may have on implementation and to assess variables within their own school context (access to jackets, how to manage the route, teachers taking part) to ensure that any adaptations do not have a negative effect on implementation.

Flexibility of delivery was highlighted as a key component in The Daily Mile’s successful implementation with relation to supporting teachers’ autonomy. Teachers were also largely engaged in the initiation of The Daily Mile at their school with their concerns addressed prior to implementation. The ability to control the daily delivery of The Daily Mile might reduce its burden on teacher’s already busy scheduled day with their engagement from the outset potentially creating a sense of ownership over the initiative. Teachers support for physical activity initiatives in schools and autonomy of delivery has previously been reported as important to implementation and sustaining interventions [22, 23]. In Scotland, The Daily Mile is being supported by the Government and subsequently many schools are keen to implement it in their setting. However, it is essential that those who will be involved in The Daily Mile (teachers and children) are involved in its initiation in order to keep what was an essentially a ‘bottom up’ approach going.

In addition to the intervention components and delivery of The Daily Mile, other aspects of how The Daily Mile was initiated also reflected in the implementation literature and whilst the intervention itself was based on the teacher’s implicit knowledge of what might work in their context, these other aspects may have been more ad hoc. Implementation science and specifically the Consolidated Framework for Implementation Research suggests that having a credible and relatable intervention source, the ability to test an intervention on a small scale prior to scaling up, and providing desired outcomes for the context are all related to implementation success [20]. From the description of how The Daily Mile began and was rolled out, many of these factors were addressed although as with many interventions developed in real life contexts, this is unlikely to have been planned or deliberate. The Daily Mile example could therefore be used as a model for future interventions on how to develop and roll out a primary school based physical activity intervention.

Other themes emerged from this study that might need to be considered for continuation of The Daily Mile. As suggested within implementation development, trialling and roll out of The Daily Mile, a supportive organisational environment was noted by all participants. Previous research suggests similar findings with an unsupportive school environment said to be related to poor implementation of school based physical activity interventions. [24, 25] For example, in a cross sectional survey of 720 principals and teachers in Canada whose schools were taking part in a comprehensive school-based programme to promote children health, including implementing 15 minutes of physical activity each day, schools who reported greater institutionalisation were two times more likely to implement the programme compared to those who reported lower levels of institutionalisation. [24] For teachers, the acute benefit of refocusing and re-energising the children appeared to be of particular importance and may contribute towards teachers continued delivery of The Daily Mile. Similar findings were reported by Holt et al. (2013) who evaluated the effect of a 20 minute physical activity policy in primary school children in the United States. [26] As with the present study, teachers in this study also noted positive classroom behaviour immediately after the physical activity had been implemented. Whilst longer term outcomes such as improved fitness or weight loss might be desirable to schools and policy makers, the acute effects provided by The Daily Mile may be more important to continued participation as these provide immediate feedback to teachers as to why the activity is worth including in their already busy day.

There are several limitations with this study including the small number of schools interviewed and only selecting schools from one geographical area. Although two schools were selected that were thought to be having challenges implementing The Daily Mile, due to a misinterpretation of what The Daily Mile was (time not distance) one school was found to be completing The Daily Mile as intended. This may have created a more biased sample towards successful implementation. In addition, only the views of key teachers involved in establishing The Daily Mile at their schools are reported. These individuals are likely to be advocates of The Daily Mile and may have a more positive view than others of the initiative. Given these limitations these results should be viewed as preliminary and further research is needed. For example, future research should speak to more schools, across different geographical areas and who have implemented The Daily Mile with varying degrees of success. The key teachers from the present study would often report what they observed and had heard from their children and colleagues. Whilst this provided a more rounded view of The Daily Mile implementation, it is also necessary to understand children’s, teachers and parent’s perceptions of The Daily Mile and specifically the compulsory aspect. This research would likely produce further and deeper insights into the implementation of The Daily Mile.

This study also provides insight into other potential future research relating to The Daily Mile. For example, whilst factors that might be related to implementation of The Daily Mile were identified through this study, without specifically testing each specific intervention factor, the extent to which these truly influence implementation is unknown and could be an area of future research. The Daily Mile was developed pragmatically without specific reference to theory. Whilst it may not be appropriate to retrospectively attach specific theories to The Daily Mile, future research may also wish to develop a program theory to help explain the behaviour change observed in children at schools undertaking The Daily Mile. This may also be applicable if looking at The Daily Mile in different contexts and future research could address whether this format is transferable to different settings such as educational institutions other than primary schools or the workplace.

## Conclusion

The Daily Mile appears to have several factors which might be related to its implementation success. This results from this study suggest that factors including having simple core intervention components, flexible delivery that supports teacher autonomy and being adaptable to suit the specific primary school context are likely to play important roles in the implementation of The Daily Mile. This research also highlights several considerations and areas of future research such as exploring exactly which of these intervention factors effect implementation and what could effects its continuation in schools. These are potentially important considerations for others looking to implement The Daily Mile effectively in their primary school or in other settings.
